# Primary malignant melanoma of the female urethra: A case report

**DOI:** 10.1016/j.eucr.2024.102909

**Published:** 2024-12-13

**Authors:** Viktoriya Boncheva, Omid Yassaie, Andrew Kennedy-Smith

**Affiliations:** Wellington Hospital, Capital & Coast, Te Whatu Ora, Wellington, New Zealand

**Keywords:** Urethral melanoma, Mucosal melanoma, Small cell type melanoma

## Abstract

Primary malignant mucosal melanoma of the female urethra is extremely rare and associated with high recurrence rates and exceptionally poor 5-year survival. Due to its rarity, treatment strategies are heterogenous and often extrapolated from the treatment of other more common types of melanomas. Herein, we describe a case of malignant melanoma of the urethra in a Caucasian female.

## 1 Introduction

Primary mucosal melanoma (MM) of the urethra is a rare malignancy which accounts for 0.2 % of all melanomas.^1^. It arises from melanocytes located within the basal cell layer of the epithelium at different anatomic sites. Melanocytes may or may not produce melanin pigmentation and can give rise to melanomas in many different locations, including respiratory, gastrointestinal and urogenital tracts.^1^. Metastatic spread occurs early in urethral melanomas, through lymphatic and haematogenous spread. These cases usually present with haematuria, obstructive urinary symptoms, protruding mass, pain and/or urethral discharge.^2^.

## 2 Case presentation

A 78-year-old woman presented to the urology outpatient clinic for a review of a rapidly growing, painful periurethral lesion. She was initially referred to and assessed by the gynaecology team who appreciated a firm mass at the anterior aspect of the vaginal introitus. The mass was reported to extend to the anterior vaginal wall, but the origin appeared to be the posterior aspect of the urethra. Her past medical history consisted of posterior neck basal cell carcinoma (excised), insulin-independent diabetes mellitus, atrial fibrillation and hypertension.

**Fig. 1:**
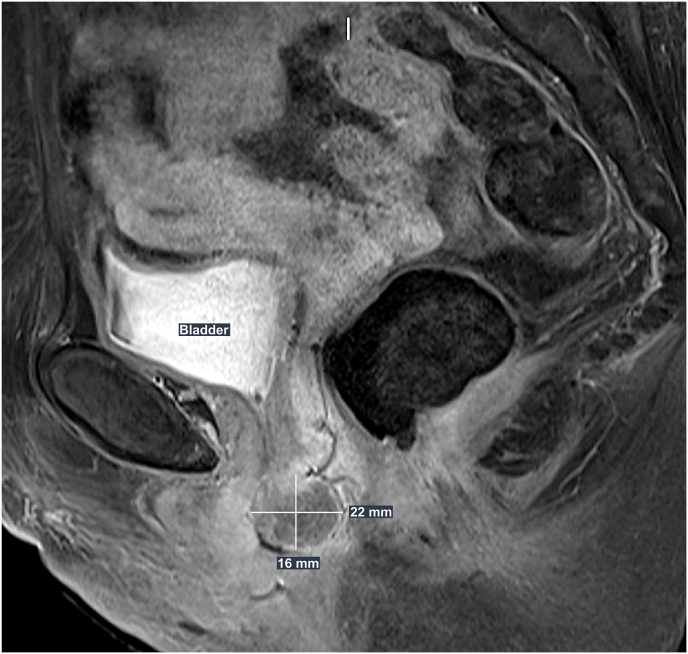
**T1-weighted MRI pelvis**. Sagittal view. A mass measuring 22mm × 16mm is identified in an intimate relationship to the inferior posterior urethra and the anterior vaginal wall. No enlarged pelvic nodes or groin nodules. This mass is concerning for malignancy.

Prior to assessment in the urology clinic, the patient underwent a Computed Tomography (CT) Urogram which did not demonstrate any intra-abdominal lymphadenopathy, urinary tract lesions or an obvious mass at the level of the urethra. However, physical examination confirmed the presence of a 3cm × 1cm solid mass at the distal posterior urethra which was tender to palpitation with no evidence of bleeding. Unfortunately, the urethral meatus was not identified due to the mass causing pain and anatomical distortion and flexible cystoscopy was not performed in the clinic. To further evaluate the nature of this lesion, a Magnetic Resonance Imaging (MRI) of the pelvis was requested. The MRI Pelvis confirmed the presence of a 22mm solid urethral meatus tumour of unknown aetiology (Fig. 1), and a decision was made to excise the lesion and reconstruct the urethra. Under general anaesthetic, the urethral meatus lesion was identified, and a rigid cystoscopy demonstrated an endoscopically normal bladder and proximal urethra.

**Fig. 2:**
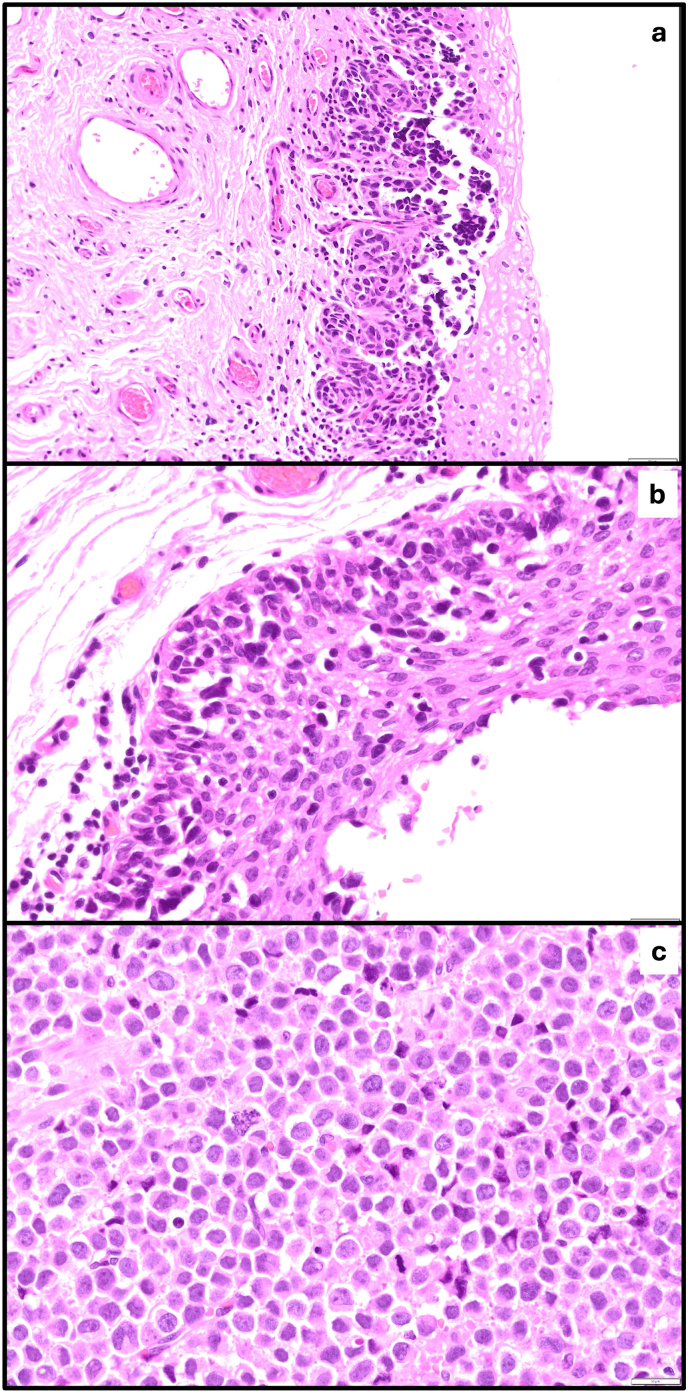
**Malignant melanoma in situ**. a) Urethral mucosa with an irregular nested proliferation of atypical melanocytes demonstrating mucosal melanoma in situ (H&E x 100). b) Urethral mucosa with an irregular nested proliferation of atypical melanocytes at the base with upward (pagetoid) extension of abnormal melanocytes, demonstrating mucosal melanoma in situ (H&E x 200). c) Cellular detail from a nodule of invasive malignant melanoma demonstrating discohesive malignant melanocytes with brisk mitotic activity and atypical mitoses (H&E x 400).

**Fig. 3:**
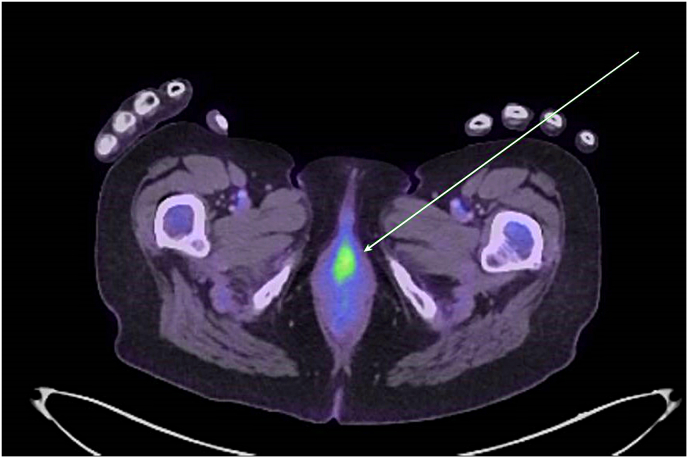
**Whole body PET-CT.** PET-CT performed post-operatively. FDG uptake around the resection site of the mid-urethra. This is consistent with residual disease/positive margins, extending over 20mm.

The lesion was successfully excised in the same manner as excision of urethral caruncle and histology revealed B-RAF negative malignant melanoma (Fig. 2a–c) with positive surgical margins. The malignant cells were immunoreactive for Melan-A and SOX 10, which are typical for melanoma. They were negative for cytokeratin, CD45, CD30 and Synaptophysin. In view of this histology, Positron Emission Tomography/CT (PET/CT) was arranged and confirmed FDG uptake at the mid urethra at the excision site (extending over 20mm) (Fig. 3) but nowhere else in the body. As per the melanoma multi-disciplinary meeting (MDM), MRI head was performed showing no evidence of metastases.

After urology MDM, melanoma MDM and patient-centred discussion, a decision was made to go ahead with anterior exenteration, cystectomy and ileal conduit formation with radiotherapy as adjuvant tool in case of positive surgical margins. The MDM recommendation was issued eight weeks following the initial excision of the urethral lesion and definitive surgery took place six weeks later. Final histology of the distal urethra showed invasive mucosal melanoma of small cell type, B-RAF negative and with clear surgical margins. Given the positive outcome, adjuvant therapy was not advised at the time. Unfortunately, the patient passed away suddenly three months after her operation, due to a stroke.

## 3 Discussion

Malignant mucosal melanoma of the genitourinary tract is an exceedingly rare and clinically aggressive type of cancer with unknown aetiology.^2^, ^3^, ^4^. Urethral melanoma is more common in the elderly, with typical median age at diagnosis of 60 years and 5-year overall survival rate of approximately 25 %. In addition, approximately two thirds of the cases are reported in women, likely due to the higher number of melanocytes in the vulvar mucosa.^5^, ^6^, ^7^. The site of melanoma occurrence in this patient was the distal urethra, which is reported to be the most common site for MM of the urinary tract.

The management of malignant MM is challenging due to the limited knowledge and lack of clear guidelines. Most common initial treatment of choice is wide excision surgery followed by surveillance CT scans at regular intervals. Unfortunately, wide excision is often associated with considerable morbidity and high local and distant metastatic recurrences, even with negative surgical margins. In the present case, we performed a simple excision in the same manner as for a urethral caruncle. The patient was subsequently referred to the local melanoma MDT and underwent anterior exenteration, cystectomy and ileal conduit formation three months after initial surgery. Given the aggressive nature of malignant mucosal melanoma, definitive surgery at an earlier stage as well as a timely referral to the melanoma MDM could have been considered. Alternatively, a wide local excision or urethrectomy could have been performed to obtain wide enough surgical margins and to prevent disease progression. However, melanoma was not suspected in this case until the histology results were available. Nevertheless, the optimum treatment for urethral melanomas in women has not yet been identified due to limited number of cases described in the literature. Therefore, it remains unclear whether a different surgical approach would have influenced her long-term outcome. Different centres/clinicians have resorted to different surgical approaches to improve survival following urethral melanoma with varying results and no consensus^8^.

Adjuvant therapy and its effects on the overall survival in this patient group also remain unclear.^3^. While the incidence of B-RAF mutations in cutaneous melanoma is quite common, it is less than 10 % in mucosal melanoma, making the use of well-established adjuvant therapy with B-RAF inhibitors ineffective. One study of 229 patients with metastatic MM found that the overall survival of patients treated with immunotherapy was longer than that of patients treated with chemotherapy.^9^. More recently, adjuvant immunotherapy with Ipilimumab, PD1 inhibitors or a combination for the management of patients with metastatic MM has become a part of the UK national guidelines^10^. However, evidence suggests that these may not be as effective as in the treatment of cutaneous melanoma and highlights the absence of MM-specific randomised studies.

Radiotherapy has been recommended for local control only, especially when surgery fails to achieve negative margins. It is also considered in the palliative setting for symptomatic or bulky lesions^10^. There is limited evidence for use of neo-adjuvant therapy in cases of MM.

Future therapeutic strategies for MM are likely to revolve around the development of mRNA vaccines which can enhance immunological responses and provide personalised therapies. A phase 2 randomized study of adjuvant immunotherapy with personalised cancer vaccine mRNA-4157 and Pembrolizumab in high-risk cutaneous melanoma is currently underway^11^. Preliminary results demonstrate reduction in the risk of disease recurrence or death in this group of patients compared to Pembrolizumab monotherapy. Personalised immunotherapy approaches like this one are likely to be beneficial in the management of MM in the future.

## 4 Conclusion

Urethral mucosal melanoma is a rare phenomenon with overall poor prognosis. Further research in this area is needed to determine the disease pathophysiology and most appropriate treatment approaches.

## CRediT authorship contribution statement

**Viktoriya Boncheva:** Writing – review & editing, Writing – original draft, Investigation, Formal analysis, Data curation, Conceptualization. **Omid Yassaie:** Supervision. **Andrew Kennedy-Smith:** Supervision.

## Funding

The research did not receive any specific grants from funding agencies.
